# Post-Consumer Textile Waste Classification through Near-Infrared Spectroscopy, Using an Advanced Deep Learning Approach

**DOI:** 10.3390/polym14122475

**Published:** 2022-06-17

**Authors:** Jordi-Roger Riba, Rosa Cantero, Pol Riba-Mosoll, Rita Puig

**Affiliations:** 1 Electrical Engineering Department, Universitat Politècnica de Catalunya, Rambla Sant Nebridi 22, 08222 Terrassa, Spain; pol.riba2@gmail.com; 2 Department of Computer Science and Industrial Engineering, Universitat de Lleida, Pla de la Massa 8, 08700 Igualada, Spain; rosa.cantero@udl.cat (R.C.); rita.puig@udl.cat (R.P.)

**Keywords:** textile waste, recycling, reuse, classification, deep learning, NIR spectroscopy, circular economy, post-consumer waste

## Abstract

The textile industry is generating great environmental concerns due to the exponential growth of textile products’ consumption (fast fashion) and production. The textile value chain today operates as a linear system (textile products are produced, used, and discarded), thus putting pressure on resources and creating negative environmental impacts. A new textile economy based on the principles of circular economy is needed for a more sustainable textile industry. To help meet this challenge, an efficient collection, classification, and recycling system needs to be implemented at the end-of-life stage of textile products, so as to obtain high-quality recycled materials able to be reused in high-value products. This paper contributes to the classification of post-consumer textile waste by proposing an automatic classification method able to be trained to separate higher-quality textile fiber flows. Our proposal is the use of near-infrared (NIR) spectroscopy combined with a mathematical treatment of the spectra by convolutional neural networks (CNNs) to classify and separate 100% pure samples and binary mixtures of the most common textile fibers. CNN is applied for the first time to the classification of textile samples. A total of 370 textile samples were studied—50% used for calibration and 50% for prediction purposes. The results obtained are very promising (100% correct classification for pure fibers and 90–100% for binary mixtures), showing that the proposed methodology is very powerful, able to be trained for the specific separation of flows, and compatible with the automation of the system at an industrial scale.

## 1. Introduction

The textile industry is fundamental for society and everyday wellbeing (i.e., comfort and protection). Different textile products are needed, such as household products and personal clothes. Textile consumption has been exponentially growing in the last decade (having doubled its production around the world in the last 15 years) due to low costs and fast fashion [1,2]. Thus, large amounts of resources are extracted to produce clothes that will last less than one year (due to being disposed of or underutilized). Therefore, there is an urgent need for the implementation of new, sustainable textile consumption patterns (e.g., textile renting), although this may not be easy [3].

Production of textile products generates great environmental impacts along their value chain, including production of raw materials, manufacturing, and the products’ end of life [1]. The main sources for raw materials in the textile industry are synthetic fibers (from oil, a non-renewable resource) and natural fibers (mostly from farming, such as of cotton, using great amounts of water and fertilizers) [4,5]. On the other hand, chemicals used during manufacturing processes (e.g., dyeing) [6] have the potential to cause environmental and human health impacts [7].

At their end of life, textile products can be reused, remanufactured, recycled, incinerated (with energy recovery), or landfilled. The degree of processing and the benefit for the environment vary from one option to the other [8]. Although material recovery should be prioritized [9], most of the textile clothes collected today are incinerated or landfilled, while a small part is reused as second-hand clothes when possible [10]. Material recovery needs a good classification method to separate the different materials for high-quality recycling.

Textile recycling rates (including reuse and remanufacture) account for only 13% of world clothing, and usually this small recycled amount is used in lower-value applications [1]. Textile industry manufacturing activities have experienced few changes in relation to traditional production systems, this being especially true in emerging countries, which account for the majority of production [11]. Recycling and reusing textiles could help to reduce the production of new textiles from raw materials and the associated use of chemicals, water, and energy in the production stage [12]. However, the shift toward a more circular textile industry still faces several challenges [13], especially in increasing the ratio of textile waste collection and recycling. Whereas textile consumption has considerably increased, textiles represent around 5% of landfill contents [14].

According to European legislation (Directive (EU) 2018/851) [15], selective collection of post-consumer textile waste should be implemented in all associated countries before January 2025, with the aim of increasing the recycling rate of textile waste in Europe. This represents a great challenge, due to the increasing complexity of textile composition [8].

Therefore, it is necessary to implement an automatic classification system to allow the correct separation of the different textile fibers, thus increasing their recyclability and the added value of recycled textile materials. This system will contribute to moving from the current linear textile system to a more circular one. Today, the classification of textiles is mainly performed manually (with high human resource costs) and rarely automated; thus, it is not possible to treat large amounts of waste each day [16].

The contributions of this paper are to the development of a sorting system able to separate the most common pure textile fibers (i.e., cotton, linen, wool, silk, polyester, polyamide, and viscose), as well as binary mixtures of viscose–polyester and cotton–polyester. The proposed sorting method is compatible with an automatic textile classification approach for textile fibers, and is based on a novel mathematical treatment (deep learning algorithms) of the near-infrared (NIR) spectra. Algorithms based on convolutional neural networks (CNNs) are applied to classify textile samples; NIR spectra can be registered through optical fiber sensors in a continuously working process, thus allowing the large-scale recycling of post-consumer textile waste. It should be noted that this approach can be applied for classifying and sorting many other materials, including plastics, paper, or leather, among others.

The near-infrared (NIR) region has a range from 750 to 2500 nm. The absorption of radiation is due to the combination of overtones and bands from the fundamental vibrations produced in the mid-infrared region [17]. These bands provide useful information, but have important limitations, such as their low intensity, which is about 2–3 times lower in magnitude compared to those in the mid-infrared. In addition, for a given molecule, different overtones and combination bands appear in the NIR region, producing a large number of overlapping bands, which makes it difficult to interpret.

Almost all of the absorption bands observed in NIR come from overtones of the stretching vibrations of AH_x_ groups or combination bands of these groups. They are mostly due to overtones of the CH, OH, and NH groups, although in some cases the bands corresponding to PH and SH groups can be observed.

The high degree of bands overlapping and the complexity of the spectrum, together with its dependence on the sample’s physical state, make it difficult to perform simple calibrations (at one or a few wavelengths) when working with the NIR technique. Thus, the use of complex mathematical methods is needed to extract useful information from the spectrum. On the other hand, its dependence on chemical and physical parameters requires the calibration to be very complete, and to take into account all of the expected variability in the samples.

One of the great advantages of NIR spectroscopy is its versatility and adaptability to analyze samples of different natures. For solid samples, the measurement is carried out by diffuse reflectance. NIR radiation has the ability to significantly penetrate a solid sample and acquire important information. This is one of the characteristics that make this form of radiation an especially useful tool in the study of solids, as chemical information can be obtained in a non-invasive and non-destructive way.

When measurements are carried out by diffuse reflectance, the use of an optic-fiber probe allows the NIR spectrum to be recorded directly on the sample, without any previous treatment, achieving a remarkable saving of time and avoiding the use of polluting reagents.

NIR spectroscopy has experienced a strong advance in recent decades, motivated both by the development of new instruments—which allow the rapid recording of the full NIR spectrum—and by the implementation of mathematical methods for the processing of spectral information. Once calibrated, the NIR spectra can be used for the fast determination of the properties of the analyzed sample [18]. Thus, this technique is being used in very diverse industrial fields, such as food, biomedicine, pharmacy, petrochemicals, polymers, and the textile industry, among others [19,20,21,22].

Thus, the aim of this paper is to develop an industrially scalable sorting method to classify textile waste (composed of pure fibers and binary mixtures) by registering the NIR spectra of the samples, followed by a mathematical treatment using convolutional neural network (CNN) algorithms.

## 2. The Proposed NIR–CNN Approach and the Processing Methods

**Figure 1:**
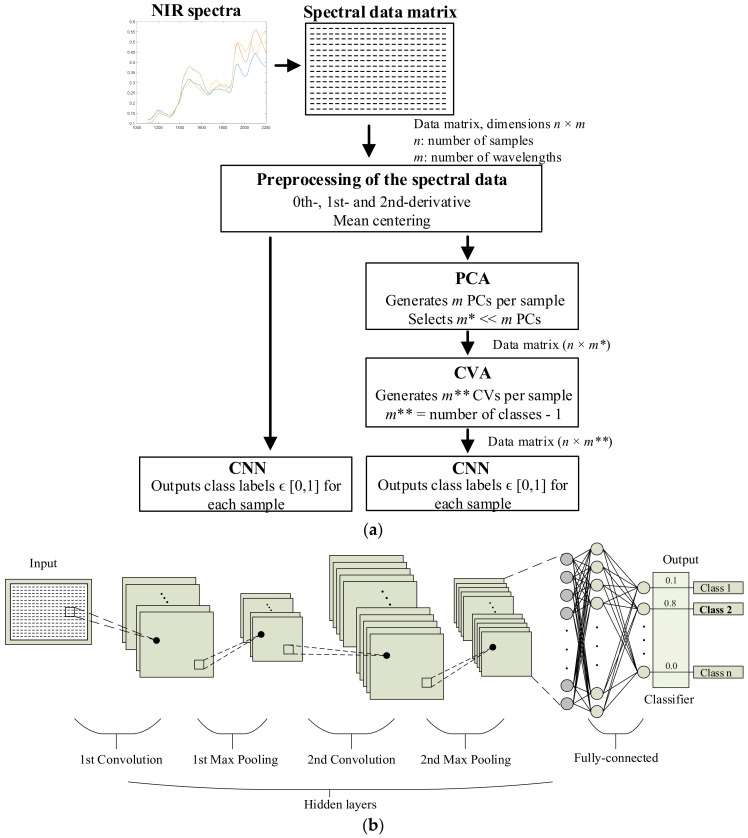
(**a**) Classification approach based on two different paths to classify an unknown fiber sample into one of the preset textile fiber classes from the NIR spectrum. (**b**) Structure of the classification dealt with by the CNN.

This paper analyzes the accuracy of two approaches for classifying unknown incoming fiber samples, based on a supervised strategy by analyzing the NIR spectral data (XDS^TM^ Optiprobe Analyzer, FOSS AnalyticalA/S, Hillerod, Denmark) of such samples using appropriate mathematical methods. These two approaches are summarized in Figure 1.

According to Figure 1, the NIR spectrum of the unknown textile sample is preprocessed by applying the first or second derivative and mean-centering the obtained data. Next, two paths are analyzed: The first consists of directly applying a convolutional neural network (CNN) to the preprocessed spectral data to classify the sample into one of the preset classes. The second path is more complex since, after the preprocessing stage, the resulting spectral data are transformed through the principal component analysis (PCA) and canonical variate analysis (CVA) algorithms, to reduce their dimensionality and prepare the data for the classification stage, which is also achieved by means of a CNN.

### 2.1. Calibration and Prediction Data Subsets

The whole dataset is often split into calibration (or training) and prediction data subsets [23]. This strategy allows assessment of the accuracy of the applied classification methods by using a different set of samples (prediction subset) than the one used to calibrate or train the model (calibration subset). It is worth noting that the prediction subset is not used during the training stage. The whole set of available data was divided in a 50%/50% proportion, i.e., half of the samples were assigned to the calibration set, and the remaining half were assigned to the prediction set. This assignment was carried out at random.

For a fair training process and a good assessment of the results attained, the pertinence class of the whole set of samples must be known. Then, the samples belonging to the calibration subset must be labelled, with the labels assigning each sample to its class (i.e., type of textile fiber), since the proposed approach is a supervised training process. Although in this paper the pertinence class of the samples in the prediction subset is already known, because it is necessary to assess the performance of the classification process, in general, the pertinence class of the prediction samples is not known, and the classification method must accurately estimate their pertinence classes.

### 2.2. Data Processing Stage

It is a common practice to process the raw spectral data provided by the spectrometer [24] to increase the accuracy of the classification stage. It should be noted that spectral data are treated in matrix format. The matrix of raw data contains n rows (the number of samples) and m columns (the number of wavelengths in each spectrum).

The usual data processing consists of obtaining the first or second derivative of the spectra. Derivatives are applied with the aim of enhancing spectral differences. They act as a specific type of baseline correction, removing constant background signals at the cost of increasing noise. Derivatives are applied for enhancing the visual resolution, highlighting spectral structures, and resolving overlapping peaks. It is known that each derivative stage decreases the polynomial order by one. Whereas the first derivative allows the removal of a constant offset, the second derivative also removes a linear term of the spectrum [25]. The derivatives were obtained by means of the Savitzky–Golay smoothing and differentiation filter, which applies a moving average of 5 points to determine the first derivative and 10 points for the second derivative of the spectra. This means that the spectral data are analyzed in raw, first derivative, and second derivative modes.

Next, the columns of the data matrix are mean-centered to remove any bias, so that afterwards the mean-centered columns have zero mean, and their variances remain unaltered.

### 2.3. Applied Dimensionality Reduction Methods

Prior to the application of the CNN it is possible to apply dimensionality reduction methods, where dimensionality refers to the number of input variables or wavelengths in the analyzed problem. These methods aim at reducing the number of variables in the problem by combining the original variables into a condensed set of new variables. This step is important because NIR spectra include thousands of wavelengths per sample, and it is known that a large number of variables can reduce the performance of classification algorithms [26].

The new variables, also known as latent variables, are usually obtained by linearly combining the original variables, with the aim of retaining the same information and rejecting the inherent noise contained in the original variables.

In this paper, the multiclass canonical variate analysis (CVA) dimensionality reduction technique is applied. It condenses the significant spectral data into a small set of latent variables [27,28] calculated by linearly combing the original variables from the NIR spectrum. The basic idea of CVA is to calculate a set of projection vectors v optimizing the Fisher criterion—that is, to maximize the differences between different classes, while minimizing the intraclass variability [29]:

(1) $$J(v_{\mathrm{CVA}})=\max\left[\frac{v^{T}{}_{\left(1,m\right)}B_{\left(m,m\right)}v_{\left(m,1\right)}}{v^{T}{}_{\left(1,m\right)}W_{\left(m,m\right)}v_{\left(m,1\right)}}\right]$$

where *v* is a vector, *m* is the number of wavelengths in the NIR spectra, and *B* is the interclass dispersion matrix, defined as follows:

(2) $$B_{\left(m,m\right)}=\sum_{i\;=\;1}^{c}n_{i}({\bar{x}}_{i}-\bar{x}){\left({\bar{x}}_{i}-\bar{x}\right)}^{T}$$

and *W* is the intraclass dispersion matrix, defined as follows:

(3) $$W_{\left(m,m\right)}=\sum_{i\;=\;1}^{c}\;\sum_{j\;=\;1}^{n_{i}}(x_{ij}-{\bar{x}}_{i}){\left(x_{ij}-{\bar{x}}_{i}\right)}^{T}$$

where ${\bar{x}}_{i}=\sum_{i\;=\;1}^{c}x_{ij}/n_{i}$, with *i* = 1,2, … *c*, *x_ij_* represents the elements of the spectral data matrix *X*_(*n*,*m*)_, *n* is the number of samples, *m* is the number of original variables or wavelengths, *c* is the number of classes defined in the problem, and $\bar{x}=\sum_{i\;=\;1}^{c}n_{i}{\bar{x}}_{i}/n$, with $n=\sum_{i=1}^{c}n_{i}$.

From the matrix of projection vectors *V*, the new latent variables or canonical variates (CVs) can be determined as *Y*_(*n*,*s*)_ *= X*_(*n*,*m*)_*V*_(*m*,*s*)_, with *s* = *c* − 1.

One of the major drawbacks of CVA is the requirement that the number of samples be greater than the number of original variables. This requirement is often not fulfilled in problems based on spectral data, since NIR spectra include thousands of variables (wavelengths), while the number of samples is usually smaller. Therefore, it is imperative to apply a suitable algorithm for reducing the dimensionality of the problem, i.e., the number of variables before CVA can be applied. To this end, principal component analysis (PCA) is applied before applying the CVA algorithm, because PCA is probably the most recognized unsupervised algorithm for dimensionality reduction [30]. PCA allows calculation of a reduced number of uncorrelated and orthogonal latent variables, known as principal components (PCs) [31]. The PCs concentrate the significant information included in the original variables [32], while explaining most of the variance in the whole set of samples. Although PCA calculates the same number of PCs as the number of original variables, only the first PCs explaining a predetermined amount of variance are retained [33], whereas the remaining PCs are not considered. To do so, the PCs must first be ranked in descending order, i.e., the first PC is that explaining the greatest variance, whereas the last PC is the one explaining the least variance. PCA is based on the singular value decomposition of matrix *X*_(*n*,*m*)_:

*svd*(*X*) = *U*_(*n*,*n*)_*Σ*_(*n*,*m*)_*V^T^*_(*m*,*m*)_ (4)

and the new latent variables can be obtained as follows:

*Y*_(*n*,*m*)_ = *X*_(*n*,*m*)_*V*_(*m*,*m*)_ (5)

By applying the PCA + CVA sequence, the dimensionality of the problem is greatly reduced, to obtain a number of latent variables (CVs) equal to the number of classes minus one. Once the CVs are calculated, the CNN algorithm can be applied to classify the unknown textile samples, although the calculation of the PCA + CVA sequence is optional.

### 2.4. Convolutional Neural Networks (CNNs) for Classification

Artificial neural networks have shown their potential for successfully predicting the structural properties of textiles [34]. CNNs are regularized, fully connected neural networks. They were first applied to recognize handwritten numbers [35]. CNNs are widely applied in image classification, financial time series, natural language processing, or to classify waste from images [36], among many other applications. In this paper, CNNs are applied to classify textile samples in different classes according to their composition. The information only flows in one direction—i.e., from the input to the output—and the number of parameters that the CNN learns is fewer than in other deep learning networks, thus reducing the computational effort.

Figure 1b shows a basic diagram of a CNN, where the inputs can be videos, images, spectra, or any other signal type, and the outputs are the probabilities of the corresponding inputs belonging to each predefined class in the problem.

CNNs usually have three main parts, i.e., an input layer, a hidden layer, and an output layer [37,38]. The hidden layers are vitally important, since they establish the relationship between the outputs and the inputs of the NN, so it is important to define the architecture according to the problem that is being addressed. The hidden layer consists of multiple sublayers connected sequentially, and its depth (i.e., the number of sublayers) depends on the complexity of the problem being addressed. As shown in Figure 1b, the typical structure of a CNN consists of a convolutional layer followed by a pooling layer (“Max Pooling” in Figure 1b), the final layer being a fully connected layer, which is connected to a classification layer in order to output values of either 0 or 1, which define the pertinence class of the input sample. 

The convolutional layer is a fundamental part of the neural network, because it extracts the relevant information from the input data [39] by applying the convolution operation between the input matrix and a filter of a given size. The output is known as the feature map. The complexity of the convolutional layer depends on the number of filters and their size. The last component of the convolutional layer is the activation function, which is applied to the feature map to avoid convergence problems. To this end, it applies a nonlinear transformation by means of an activation function, such as the sigmoidal, hyperbolic tangent, rectified linear unit (ReLU), or Maxout functions. The pooling layer often follows the convolutional layer. It is applied to reduce the size of the data coming from the convolutional layer, thus reducing the computational effort and improving the classification accuracy [39]. Finally, the fully connected layer is where the classification process takes place. Neurons in a fully connected layer are connected to all activations in the previous layer. The fully connected layer compiles the data extracted by the preceding layers to generate the final output. 

A CNN requires a specific data structure, so it is necessary to accommodate the input data to the requirements of the CNN. Therefore, the input data are fed to the CNN as a matrix, where the columns are the wavelengths of the spectral data, while each row corresponds to a different sample. The output of the CNN is a classification matrix, where every column corresponds to the probability of the sample belonging to each of the defined classes, and every row refers to a different sample.

The next step is to define the structure of the CNN. In this case, two convolutional layers with the subsequent pooling layers were used, as shown in Figure 1b, as a compromise between computational effort and accuracy. The fully connected layer has as many outputs as the number of classes defined in the problem. In this case, the fully connected layer was followed by a classification layer to ensure that only one of the output values was 1, with the others being 0.

### 2.5. Training of the CNN

Two convolutional layers were selected after training CNNs with different numbers of layers, this solution offering a good balance between computational burden and accuracy [40]. The Adam solver was selected because it offers accurate results, since it efficiently updates the neural network weights [41]. The results of the training process are also highly dependent on the values of the different parameters of the CNN. To solve this issue, an optimization approach was considered by applying the Bayesian optimization algorithm (BOA) [42], because this requires fewer iterations in comparison with traditional optimization algorithms. The BOA initially assumes a prior distribution model of the parameters’ function, and uses the data obtained to optimize the training model. The BOA finds the values of the parameters in a bounded domain to improve the result by finding a global optimum (which minimizes the training error) using the information of the previous analyzed point [43]. The BOA was applied to determine the optimal values of the number of epochs (NE), the validation frequency (VF), the validation patience (VP), the verbose frequency (VFreq), the number of filters in the first layer (NF_1st) and the number of filters in the second layer (NF_2nd) during the training process. The BOA function implemented in MATLAB^®^ automatically stops when reaching any of the three following conditions: reaching a fixed number of iterations (30 by default), a fixed time (no time limit by default), or a stopping criterion.

## 3. Sample Collection and Identification

This paper analyzes textile fibers obtained from several catalogs of different companies. The analyzed samples (textile fibers) included natural, synthetic, and artificial fibers, as well as combinations of natural and synthetic/artificial fibers in different proportions. Natural fibers include silk, wool, linen, and cotton. Synthetic fibers include polyamide and polyester, while viscose is an artificial fiber. While artificial fibers are generated by transforming natural products (e.g., viscose comes from cellulose), synthetic fibers are generated from petrochemical polymers. Hereafter, artificial and synthetic fibers will be referred to as manmade fibers.

A sequence of three studies was designed. For each study, the selected textile fibers were chosen to include maximum variability, so different presentations (fabric or yarn) and a multitude of colors (from dark to light) were included in the analyzed set of samples. The three studies are described below (see Table 1):

- Study #1: To classify 100% pure textile samples among seven different classes (i.e., cotton, linen, wool, silk, polyester, polyamide, and viscose).
- Study #2: To classify mixtures of viscose and polyester in different percentages.
- Study #3: To classify mixtures of cotton and polyester in different percentages.

Samples were provided by different companies (a total of eight) in a span of 4 years (2016 to 2019). A total of 52 different commercial catalogues together with additional spare samples provided by the companies was used to obtain the resulting samples for each of the three studies. The number of catalogues used in each study is presented in Table 1, as well as the number of samples per class and per study. The total number of samples used (considering the three studies) was just above 300. Samples could not be classified visually because their appearance varied widely. The composition of each sample is assured because it is exactly defined in the catalogue provided by the company.

## 4. The Analyzed Spectral Data

This study is based on the NIR spectral data of the analyzed textile fibers. To acquire the NIR spectra of the studied samples, an NIR spectrometer equipped with a reflectance fiber-optic module (FOSS XDS^TM^ OptiProbe Analyzer provided by FOSS AnalyticalA/S, Hillerod, Denmark.) controlled by the Vision Software^TM^ was used. Although this was able to acquire the spectra in the 400–2500 nm range, the 1100–2200 nm range was analyzed to reduce the effect of noise and to avoid the visible region. To further reduce the effect of noise, the spectral data were acquired through averaging of 32 scans. Since the resolution of the FOSS XDS^TM^ OptiProbe Analyzer is 0.5 nm, each spectrum has 2201 points per sample. Next, the raw data provided by the NIR spectrometer were processed and transformed by means of the mathematical methods detailed in Section 2.

**Figure 2:**
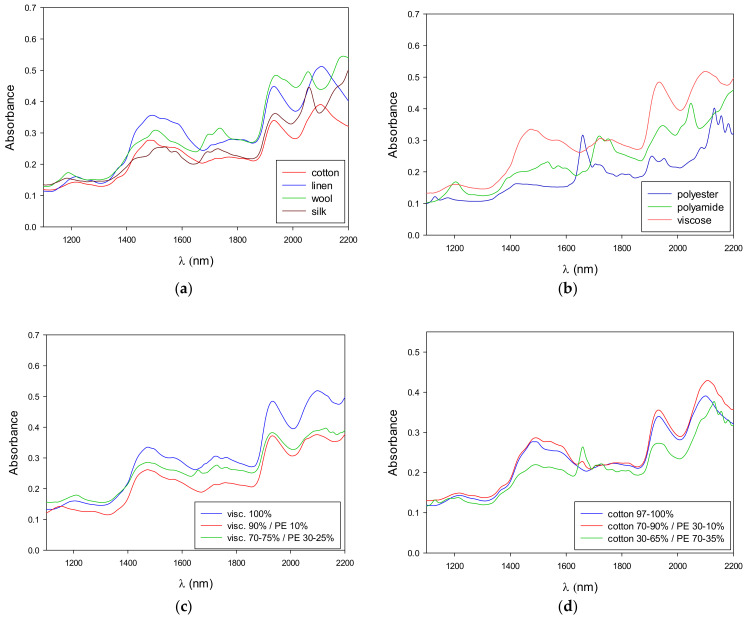
(**a**) Natural fibers. (**b**) Manmade fibers. (**c**) NIR spectra of binary mixtures (viscose/PE). (**d**) NIR spectra of binary mixtures (cotton/PE).

The NIR spectra of some of the samples analyzed are shown in the following figures. Figure 2a shows the NIR spectra of natural fibers (i.e., cotton, linen, wool, and silk), while manmade fibers are presented in Figure 2b. The similarities and differences between those samples in different ranges of the NIR spectrum can be observed (i.e., the similarity between cotton and linen is high, with both of them also being similar to viscose, while the synthetic fibers are very different from them). 

There were three main characteristic bands for cotton: one around 1480 nm, the second at 1942 nm, and the third at 2100 nm. A similar pattern was observed for linen and viscose, as both are cellulose-based fibers, like cotton. In the case of polyamide, a peculiar band was observed at 1713 nm, which corresponds to combination overtones of NH bonds from the amide functional group [44]. This band was also observed in wool and silk (due to amide functional groups from proteins). Finally, the most distinctive band for polyester was found at 1661 nm.

In addition, spectra from binary mixtures of fibers (viscose/PE and cotton/PE) are shown in Figure 2c,d, respectively. In this case, the differences are more difficult to identify at first sight. As noted above, the NIR spectra exhibit wide bands, overlapped bands, and overtones, making it difficult to interpret the spectral information. Therefore, mathematical treatment of the spectra is needed. Nevertheless, we can clearly observe in Figure 2d that, when increasing the amount of PE in the binary blend, the characteristic band of this fiber (at 1661 nm) grows.

## 5. Experimental Results

In this section, three studies with different textile samples are presented to prove the accuracy of the proposed approach.

### 5.1. Study #1: Analyzing Pure Textile Fibers

This first study analyzed 210 pure textile samples, i.e., the samples were made of pure fibers, as summarized in Table 1.

As shown in Table 2, Study #1 considered seven classes of fibers; thus, the goal was to correctly classify all samples of the prediction set, which was composed of 15 × 7 = 105 samples. Two approaches were assessed to this end: the first was based on directly applying the CNN to the raw spectral data, or to the first or second derivatives, and mean-centering the data; whereas the second alternative applied the PCA + *CVA* sequence prior to the CNN, as described in Figure 1. 

Table 2 summarizes the classification results attained with the two approaches, as well as the CNN and PCA parameters selected in each test. These parameters are the number of epochs (NE), validation frequency (VF), validation patience (VP), verbose frequency (VFreq), the number of filters in the first layer (NF_1st), and the number of filters in the second layer (NF_2nd). Finally, we retained a number of PCs explaining more than 99.99% of the total variance.

The results in Table 3 clearly show the better performance of the PCA + CVA + CNN approach compared to the performance of the CNN alone. This implies that the PCA + CVA feature-selection stage optimally prepares the data for the classification phase, thus simplifying the task of the classifier, greatly reducing the dimensionality of the problem and the computational burden during the training stage, since the dimensionality was reduced from 2201 variables to only 6 in this study (i.e., the number of classes minus one). Therefore, the subsequent studies were based only on PCA + CVA + CNN, due to the superior results obtained by means of this approach. The results summarized in Table 3 also prove that the PCA + CVA + CNN approach enables us to correctly classify the 105 samples of the prediction set. 

### 5.2. Study #2: Analyzing Mixed Viscose–Polyester Textile Samples

This second study analyzed 73 mixed textile samples, which included pure viscose and viscose/PE mixtures, as summarized in Table 4.

As shown in Table 4, Study #2 considered three classes of fibers; thus, the goal was to correctly classify all samples of the prediction set, which was composed of 13 × 2 + 10 = 36 samples. Due to the superior results, Study #2 only contemplated the PCA + CVA + CNN approach. 

Table 5 summarizes the classification results attained with the PCA + CVA + CNN approach, as well as the CNN and PCA parameters selected in each test. 

As seen in Table 5, when applying the first and second derivatives to the spectral data, the PCA + CVA + CNN method correctly classifies the 36 samples of the prediction set. 

### 5.3. Study #3: Analyzing Mixed Cotton–Polyester Textile Samples

This third study analyzed 90 mixed textile samples, which included pure cotton and cotton/PE mixtures, as summarized in Table 6.

As shown in Table 6, Study #3 considered three classes of fibers; thus, the goal was to correctly classify all samples of the prediction set, which was composed of 15 × 3 = 45 samples. Due to the superior results, Study #3 only contemplated the PCA + CVA + CNN approach. 

Table 7 summarizes the classification results attained with the PCA + CVA + CNN approach, as well as the CNN and PCA parameters selected in each test. 

As seen in Table 7, the PCA + CVA + CNN method correctly classifies between 38 and 41 out of the 45 samples of the prediction set. Some of the misclassified samples are used to make shoes, thus presenting a particular finishing type.

## 6. Conclusions

The main conclusion of the present work is that the developed method (using a near-infrared (NIR) sensor and mathematical treatment based on convolutional neural networks) allows us to correctly classify pure textile fibers (i.e., cotton, linen, wool, silk, polyester, polyamide, and viscose) and binary blends (i.e., viscose/polyester or cotton/polyester).

The results in Table 3 clearly show the better performance of the PCA + CVA + CNN approach compared to the performance of the CNN alone. This implies that the PCA + CVA feature-selection stage optimally prepares the data for the classification phase, thus simplifying the task of the classifier, greatly reducing the dimensionality of the problem and the computational burden during the training stage, since the dimensionality is reduced from 2201 variables to only 6 or 2 in this study (i.e., the number of classes minus one).

The best classification was obtained with the consecutive application of the PCA + CVA + kNN methods, achieving 100% correct classification rates for pure samples in absorbance mode, 100% correct classification rates for viscose/polyester blends (viscose/PE) in first derivative mode, and 91.1% for cotton/polyester blends (cotton/PE) in second derivative mode.

It should be remembered that in the latter case the three classes defined for the cotton/polyester blends (cotton/PE) were very broad and similar: cotton > 97%; cotton 70–90%; and cotton 30–65%. In contrast, the viscose/PE mixtures were three narrower groups, and were more separated from one another: viscose 100%; viscose 90%; and viscose 70–75%.

The method is powerful, and can be trained to separate pure fibers and the most interesting binary mixtures; its only limitation is the need for a sufficiently large database with samples of known composition. This can help the textile industry to be more circular, by obtaining secondary textile polymers and reducing the need for natural (i.e., crop- or animal-based) and synthetic (i.e., oil-based) virgin materials.

## Figures and Tables

**Table 1:** Origin and number of samples used in each study.

Studies	Catalogs (n°)	Classification Classes	Samples per Class	Samples per Study
Study #1	25	Cotton; linen; wool; silk; polyester; polyamide; viscose	30	210
Study #2	11	Viscose (100%);	26	73
		viscose/PE (90%/10%);	26	
		viscose/PE (70–75%/30–25%)	21	
Study #3	25	Cotton (>97%);	30	90
		cotton/PE (70–90%/30–10%);	30	
		cotton/PE (30–65%/70–35%)	30	

**Table 2:** Study #1: Pure textile samples.

Pure Fiber	Type of Fiber	Samples Number		
		Calibration	Prediction	Total
Cotton	Natural	15	15	30
Linen	Natural	15	15	30
Wool	Natural	15	15	30
Silk	Natural	15	15	30
Polyester (PE)	Synthetic	15	15	30
Polyamide (PA)	Synthetic	15	15	30
Viscose	Artificial	15	15	30

**Table 3:** Study #1: Prediction set classification errors (105 samples).

Conditions	Classification Errors	
	CNN	PCA + CVA + CNN
Mean-centering	4/105 ^a^	0/105 ^aa^
First derivative + mean-centering	3/105 ^b^	0/105 ^bb^
Second derivative + mean-centering	4/105 ^c^	1/105 ^cc^

^a^ NE = 200, VF = 8, VP = 29, VFreq = 10, NF_1st = 8, NF_2nd = 16. ^b^ NE = 500, VF = 18, VP = 20, VFreq = 10, NF_1st = 9, NF_2nd = 14. ^c^ NE = 385, VF = 18, VP = 20, VFreq = 10, NF_1st = 9, NF_2nd = 12. ^aa^ NE = 300, VF = 1, VP = 30, VFreq = 1, NF_1st = 8, NF_2nd = 16, 14 PCs. ^bb^ NE = 600, VF = 9, VP = 10, VFreq = 9, NF_1st = 6, NF_2nd = 18, 45 PCs. ^cc^ NE = 500, VF = 24, VP = 30, VFreq = 28, NF_1st = 10, NF_2nd = 16, 64 PCs.

**Table 4:** Study #2: Pure viscose and viscose/PE mixtures.

Pure Fiber	Composition	Samples Number		
		Calibration	Prediction	Total
Viscose	100% (Pure)	13	13	26
Viscose/PE	90%/10%	13	13	26
Viscose/PE	70–75%/30–25%	10	11	21

**Table 5:** Study #2: Prediction set classification errors (36 samples).

Conditions	Classification Errors
	PCA + CVA + CNN
Mean-centering	1/36 ^a^
First derivative + mean-centering	0/36 ^b^
Second derivative + mean-centering	0/36 ^c^

^a^ NE = 605, VF = 1, VP = 13, VFreq = 7, NF_1st = 5, NF_2nd = 1, 8 PCs. ^b^ NE = 200, VF = 4, VP = 15, VFreq = 4, NF_1st = 6, NF_2nd = 18, 27 PCs. ^c^ NE = 327, VF = 22, VP = 12, VFreq = 7, NF_1st = 6, NF_2nd = 12, 32 PCs.

**Table 6:** Study #3: Pure cotton and cotton/PE mixtures.

Pure Fiber	Composition	Samples Number		
		Calibration	Prediction	Total
Cotton	≥97% (Pure)	15	15	30
Cotton/PE	70–90%/30–10%	15	15	30
Cotton/PE	30–65%/70–35%	15	15	30

**Table 7:** Study #3: Prediction set classification errors (36 samples).

Conditions	Classification Errors
	PCA + CVA + CNN
Mean-centering	7/45 ^a^
First derivative + mean-centering	7/45 ^b^
Second derivative + mean-centering	4/45 ^c^

^a^ NE = 500, VF = 21, VP = 28, VFreq = 5, NF_1st = 8, NF_2nd = 16, 12 PCs. ^b^ NE = 300, VF = 7, VP = 6, VFreq = 10, NF_1st = 8, NF_2nd = 16, 27 PCs. ^c^ NE = 404, VF = 4, VP = 29, VFreq = 23, NF_1st = 8, NF_2nd = 16, 42 PCs.

## Data Availability

Not applicable.
